# Recent Progress on Natural Fibers Mixed with CFRP and GFRP: Properties, Characteristics, and Failure Behaviour

**DOI:** 10.3390/polym14235138

**Published:** 2022-11-25

**Authors:** Ariyana Dwiputra Nugraha, Muhammad Irfan Nuryanta, Leonard Sean, Kresna Budiman, Muhammad Kusni, Muhammad Akhsin Muflikhun

**Affiliations:** 1 PLN Research Institute, Jakarta 12760, Indonesia; 2 Mechanical and Industrial Engineering Department, Gadjah Mada University, Jl. Grafika No. 2, Yogyakarta 55281, Indonesia; 3 Faculty of Mechanical and Aerospace Engineering, Bandung Institute of Technology, Jalan Ganesa 10, Bandung 40132, Indonesia; 4 Center for Advanced Manufacturing and Structural Application (CAMSE), Gadjah Mada University, Jl. Grafika No. 2, Yogyakarta 55281, Indonesia

**Keywords:** natural fibers, GFRP, CFRP, mechanical properties, composite manufacturing, failure analysis

## Abstract

Research on natural-fiber-reinforced polymer composite is continuously developing. Natural fibers from flora have received considerable attention from researchers because their use in biobased composites is safe and sustainable for the environment. Natural fibers that mixed with Carbon Fiber and or Glass Fiber are low-cost, lightweight, and biodegradable and have lower environmental influences than metal-based materials. This study highlights and comprehensively reviews the natural fibers utilized as reinforcements in polyester composites, including jute, bamboo, sisal, kenaf, flax, and banana. The properties of composite materials consisting of natural and synthetic fibers, such as tensile strength, flexural strength, fatigue, and hardness, are investigated in this study. This paper aims to summarize, classify, and collect studies related to the latest composite hybrid science consisting of natural and synthetic fibers and their applications. Furthermore, this paper includes but is not limited to preparation, mechanism, characterization, and evaluation of hybrid composite laminates in different methods and modes. In general, natural fiber composites produce a larger volume of composite, but their strength is weaker than GFRP/CFRP even with the same number of layers. The use of synthetic fibers combined with natural fibers can provide better strength of hybrid composite.

## 1. Introduction

Composite materials with high specific stiffness and strength have been attracting increased interest from the automotive sector because of their lightweight structural properties. Composite materials are employed in various fields, such as aviation, shipping, building, chemical, and transportation industries. Composite materials are also extensively used in many products from macro size to nano size, such as propeller blades, aircraft frame structures, boats, yachts, cisterns, car hoods, nano composite materials, and residential refrigerators [1,2,3,4]. Composite materials are widely used because they have a low density, superior mechanical properties, fatigue endurance and toughness, versatility and well-tailored design, and a straightforward manufacturing process [5,6]. In general, composites can be made with natural fiber that require less energy, recyclable, low production costs, and exhibit good environmental sustainability [7]. With the developments in research, natural fibers have been utilized as reinforced composite materials in different areas. These materials are light and readily available in nature. The significant advantages of these materials are their renewability and biodegradability [8]. When the components of natural fiber composite are all biobased, composites can rapidly decompose naturally in the environment at the end of their life cycle [9]. Composite material also can be produce with synthetic fibers, such as glass, carbon, aramid, and kevlar [10,11,12]. Composites often use synthetic fibers as reinforcement. In terms of cost, carbon fiber is the fiber with the highest price, but it is comparable to the good mechanical properties provided. The relatively higher price of carbon fiber than glass fiber makes it used to manufacture crucial components that require resistance to high loads. The economic value obtained from glass fiber has developed regarding material modification. One of the developments carried out is by combining it with natural fibers to create a hybrid composite. In the future, natural fibers offer advantages to be used as renewable materials that are very promising because of their lower density than synthetic fibers, which affect the weight of the composite, so that it will conserve energy if applied to the automotive industry.

The biodegradability of natural fiber protects the environment from plastic materials, and its relatively low price makes it attractive to various industries [13]. As a natural fiber that contains hydroxyl groups, making it difficult to bond with the hydrophobic matrix, surface treatment on natural fibers needs to be done to increase the interaction with the matrix [14]. Composite with natural fiber reinforcement have replaced many conventional materials in several fields and have attracted considerable attention because of their outstanding characteristics compared with other materials [15]. Moreover, natural fiber is nonabrasive and relatively low price compared with traditional glass or carbon fibers, resulting in a competitive price/performance ratio in large-volume engineering markets [16]. The use of natural fibers as hybrid composites with specific fractions can provide suitable dielectric constants and dissipation factors so that they can be used as dielectric materials, such as microchips, terminals, connectors, and circuit boards [17]. Natural fiber composites produce less emissions and no abrasion during processing. Natural fibers that can be used as reinforcement include sisal, bamboo, jute, cotton, and ramie [18].

Natural fiber composites have been developed and applied in the manufacture of car parts, such as door panels, dashboards, car bottom panels, [19]. airplane components and most engineering fields [20]. Composites with natural fiber reinforcement also increasingly being applied in sports equipment, construction, electronics, and food packing industries [21]. Hemp fiber has the most potential for use in natural composites because of its superior mechanical properties [22]. To improve the strength of natural fiber, specific treatments, for example, alkaline treatment, which can produce higher mechanical properties, improve the flexural and young’s moduli, [23,24]. Treatment by modifying the surface of fiber positively correlates with the interaction between fiber and matrix [25].

**Figure 1:**
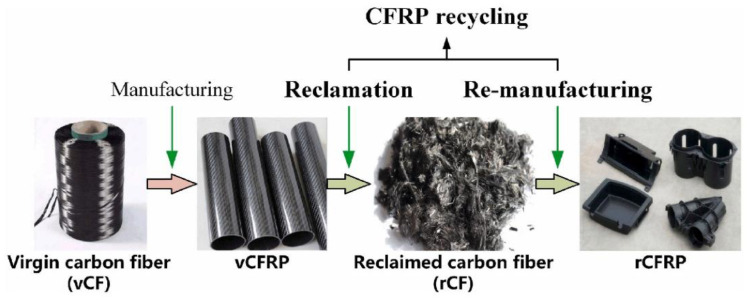
Utilization of used carbon fiber (modified from [33]).

Another concern related to the use of natural fiber combined with CF or GF was about recycle issue and environmental aspects that directly linked to emission and pollution. Environmental problems related to the use of GFRP/CFRP have driven researchers to make these materials more environmentally friendly. The development of CFRP is hindered because it produces waste that is difficult to decompose, especially composite materials with thermoset matrix [26]. Regarding the matrix type, composites with thermoplastic matrices have better natural degradability [27]. Cross-linked chemical bonds make the thermosets matrix more involved in recycling [28]. The study that has been carried out to minimize GFRP waste occurred by converting fiber into powder to be used as a filler such as in the asphalt matrix composite. This offers advantages including cost savings, efficiency and material resistance [29]. Reusing carbon or glass fiber as the core structural materials is not recommended due to the degradation of fiber quality that occurs because of the chemical factors in the adhesion level that not optimal as the new CFRP or GFRP laminates [30,31,32]. For the secondary structure, recycled carbon fiber or glass fiber can be used. The research that combined carbon fiber waste with PLA as shown in Figure 1 was successfully applied. The specimen was printed in the form of a tensile test with 3D printing, the test results showed that using recycled carbon fiber could increase the tensile strength [33]. Besides improving material properties, synthetic fiber recycling must be cost-effective [34,35]. The degradation process of synthetic fibers naturally depends on environmental conditions. Recycling matrix and fiber have a significantly positive impact, such as maintaining nature and saving energy. However, composite materials at risk condition often end up in landfills or incineration [36,37,38,39]. Replacement or hybridization of synthetic fibers with natural fibers is one option to maximize the degradation ability of composites.

Inspired by the development of hybrid composite technology consist of natural and synthetic fibers, this paper summarizes, classifies, and collects studies related to the latest composite hybrid science consisting of natural and synthetic fibers and their applications. The natural fibers discussed in the present study were jute, bamboo, sisal, kenaf, flax, and banana that combined with carbon fiber (CF) and glass fiber (GF). The review studies described in this paper range from preparing the hybrid composite and the mechanism to evaluating the tests that conducted in different methods and modes.

## 2. Data Source

Data sources are searched using several research data service providers, such as Google Scholar and ScienceDirect. In addition to the literature, we retrieved and analyzed academic citations using Harzing’s Publish or Perish software. We selected sources from the Scopus database from 2018 to 2022. We retrieved several articles that matched the topic for the review process. The papers selected in the present study were based on those highly cited by researchers and listed in reputable databases (i.e., Scopus and Web of Science). The selection of literature must comply with the following: (1) the language used in the literature must be English, (2) the literature have standard term of academic paper, while the paper that only have abstract are excluded, (3) the literature must focus on the study of (“natural fibers” OR “natural composites” OR “hybrid composites” OR “composite fabrication”) AND (“fiber properties” OR “fiber characteristics” OR “composite properties” OR “Jute hybrid laminates” OR “Bamboo hybrid laminates” OR “Sisal hybrid laminates” OR “Kenaf hybrid laminates” OR “Flax hybrid laminates” OR “Banana hybrid laminates”). Article types were filtered to “review articles” and “research articles.” The appropriate literature is grouped into several discussion topics by examining the title and abstract. The articles obtained can be used as a reference to expand the relevant literature. After the selection, other journal databases were also searched using Google Scholar to check and recheck if other papers have an impact on the study and provide useful information to strengthen the present study.

## 3. Results

### 3.1. Properties of Mixed Fibers

A few papers discussing different kinds of hybrid natural fiber composites with carbon or glass fiber are shown in Table 1.

### 3.2. Jute Fiber Hybrid Composites

#### 3.2.1. Jute-GFRP

Glass/jute hybrid composites are manufactured using the hand layup method with different stacking sequences based on the study by Khalid et al. [56]. The hybridization method simultaneously addresses the manufacturing issues and partially supplies biomaterials. The stacking sequence is significantly affected by the mechanical properties of hybrid composites. Because of its significant impact properties, jute can be utilized in ballistic armor applications more than other natural fibers. The tensile test was conducted according to the ASTM D3039 standard. The load and environment were considered in the typical tensile stress test. Four samples from each composite laminate were examined. The three best sampling results for each laminate are presented in compressive deformation diagrams. The results confirmed that the G/G/G/G/G sequence has the best tensile strength of 87 MPa. The hybrid composite with the G/J/J/J/G sequence has the worst tensile strength among all laminates examined because these hybrid laminates include a high proportion of jute fibers. The properties of the composite are significantly inferior to those of glass fibers. Microvoids, poor fiber interaction, and adhesion failure between individual fiber layers can trigger the delamination of the laminates. These aspects are the main results of the fractographic investigation of these specimens. Jute fiber is widely used in the agriculture, textile, woven, and nonwoven sectors because of its relatively low price. However, this fiber has meager crease resistance and is prone to mechanical strength degradation when wet [57,58].

**Figure 2:**
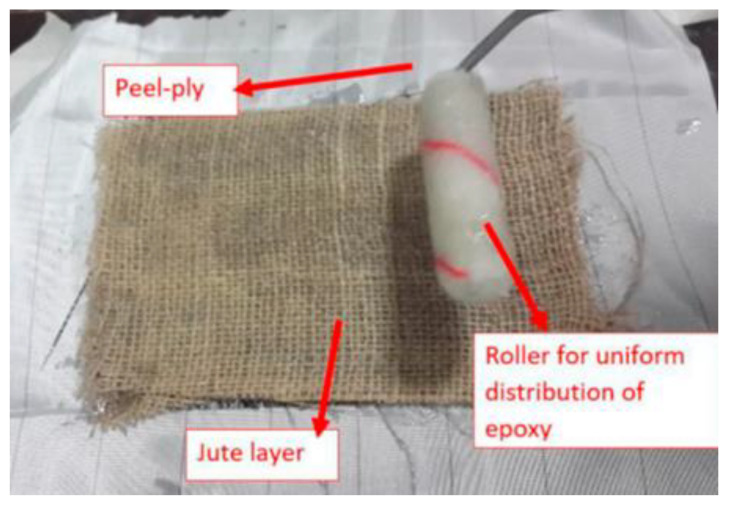
Jute/glass hybrid composite manufacture (modified from [56]).

The use of the hand layup technique in the manufacturing process is relatively easy and low-cost [59]. First, wax is applied to the mold so that the resin can be easily removed. Then, the fiber layers are arranged according to the specified variable. A roller is used to flatten the resin and remove air bubbles. The use of peel ply aims to facilitate the release of the composite. Figure 2 shows the tools and materials used to manufacture composites using the hand layup technique.

**Figure 3:**
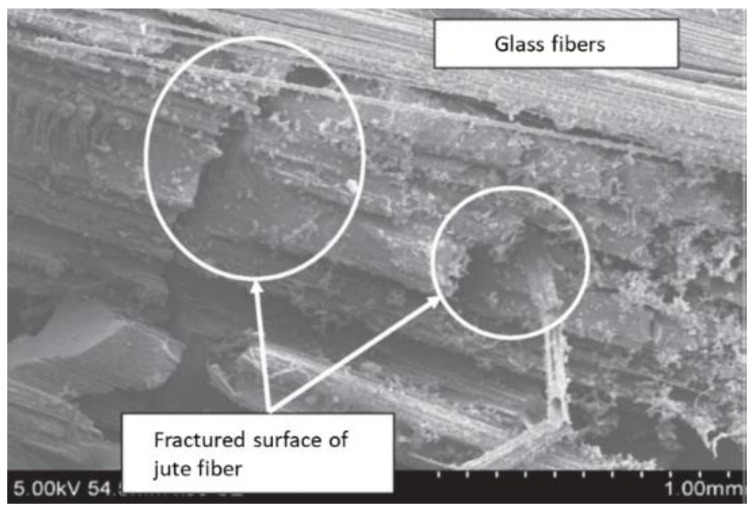
SEM image of Jute-GFRP sample after tensile test (modified from [60]).

Natural fibers have remarkably increased because the application sector, particularly the automobile industry, has been expanding every day. Jute fibers are low-cost with broad applications in the agriculture, textile, woven, and nonwoven sectors. However, these fibers experience a reduction in their mechanical strength when wet, thereby leading to meager crease strength [56]. Ramesh et al. [59] determined whether sisal/jute/glass composite is a viably low-cost, sustainable option. In the tensile and bending tests, a universal testing machine subjects the sample to a certain load until deformation or break occurs. The highest tensile strength was exhibited by jute/GFRP (229.54 MPa), followed by sisal/jute/GFRP (200 MPa) and sisal/GFRP (176.20 MPa). In the flexural test, the highest strength was exhibited by the sisal/jute/GFRP composite (3 kN), followed by sisal/GFRP (2.3 kN) and jute/GFRP (2.1 kN). Morphological observations of the jute/GFRP samples were conducted using scanning electron microscopy (SEM), as shown in Figure 3. Observations of the fracture conditions indicate the failure of the fiber under a certain load, which causes the fiber strength to decrease.

**Figure 4:**
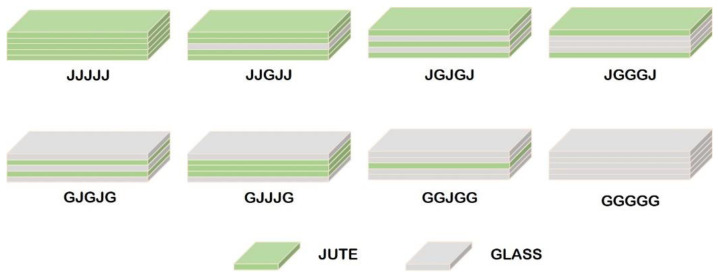
Layer configuration specimens (modified from [61]).

A study on the effect of layer configuration was carried out by Ghani et al. [61]. Hybrid composites were made by combining jute and glass fibers. The arrangement of the layers used can be seen in Figure 4. Composite manufacture uses compression molding technique to determine its mechanical strength, and tensile and bending tests. The strength of the hybrid composite is very influential on the composition of its constituent fibers. The result is that providing an arrangement of glass fibers on the outer part of the composite (GGJGG) can increase its mechanical strength.

Kennedy et al. [62] discussed how different stacking sequences can be used to determine the mechanical properties of glass/jute composites. Two glass piles on both sides lead to high costs and enhanced properties. Layers of fibers are stacked and laminated with resin and arranged in four different combinations. Both glass and jute fibers were stacked in two ways: alternating glass and jute fibers (Hybrid 1) and six layers of jute fiber followed by six layers of glass fiber (Hybrid 2). The samples were tested through performance analysis. Radiographic testing was conducted to identify good specimens for the subsequent tests to increase the accuracy of the results. The tensile test showed that the composite with the highest tensile strength is glass-only composite (228 kN), followed by Hybrid 2 (89 kN), Hybrid 1 (47 kN), and jute-only composite (26 kN). The interfacial bond between fiber and resin significantly affects the hardness of the composite. In the impact test, Hybrid 2 exhibited a better performance in sustaining impact than Hybrid 1.

Furthermore, bamboo/jute/glass fiber composites were successfully tested by Chandramohan et al. [63] with polyester as matrix and bamboo, glass, and jute as reinforcements with the unidirectional lamina. The glass/jute composite results were recorded with the different analyses. Seven composites with varying arrangements of layer were manufactured using the limited layup technique. The order in which the composite layers are arranged is presented in Table 2. After curing, the specimens were released from the composite sheets, consistent with the ASTM D3039 standard for tensile tests, ASTM D790 standard for flexural tests, and ASTM D5379 standard for shear tests. The lowest tensile strength values of various advanced composites have been determined for four layers of jute fiber composite (34.87 MPa under dry conditions) and G/J/J/J/G (51.41 MPa under dry conditions). The flexural strength of four layers of jute fiber composite is 67.56 MPa, whereas that of G/J/J/J/G is 92.32 MPa (both under dry conditions). The shear strength of J/J/J/J/J is 68.94 J, whereas that of G/J/J/J/G is 81.41 J. Overall, the glass/jute composite performs better than the jute-only composite.

#### 3.2.2. Jute-CFRP

The use of jute-FRP in structural retrofitting was investigated by Sen and Reddy [64]. The materials used were jute fiber and CFRP/GFRP composites. The results showed that the composite with jute fiber exhibited a tensile strength value of 189,479 N/mm^2^, whereas the composites with glass fiber and carbon exhibited tensile strength values of 678,571 N/mm^2^ and 923,056 N/mm^2^, respectively. The flexural strength value of the composite with jute fiber was 208,705 N/mm^2^, that of the composite with glass fiber was 666,871 N/mm^2^, and that of the composite with carbon fiber was 1,587,134 N/mm^2^. In conclusion, the jute-only composite is not powerful enough on its own. Reinforcement of glass and carbon is needed to compete against CFRP and GFRP.

**Figure 5:**
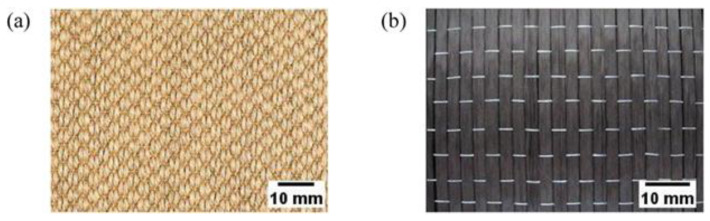
(**a**) Jute and (**b**) carbon fibers (modified from [66]).

The study conducted by Ashworth et al. [65] focused on combining glass and carbon with natural fibers under internal pressure, leading to a desirable mix of performance, environmental effect, and cost production. The materials used were 199 gsm twill 3K-T300 carbon fiber fabric and 550 gsm twill jute fabric, which were cut into 28 cm × 28 cm plies and mixed with epoxy and hardener. A 300 × 300 × 3 mm mold panel was fabricated for the RTM process (Figure 5). Each layer of fiber stacked was first subjected to 4 bar pressure and then 8 bar pressure with resin. Then, it was left to cure and sealed. Their results showed that CFRP (535.2 MPa at 8 bar) outperformed natural-fiber-reinforced polymer (NFRP; 45.5 MPa at 8 bar) in terms of tensile strength. The hybrid composite C/J/J/J/C exhibited a good tensile strength of 92.4 MPa at 8 bar. In conclusion, the carbon/jute hybrid is a sustainable replacement option for the more costly CFRP.

Sujon et al. [66] analyzed jute and carbon (Figure 5) as reinforcements and epoxy resin as a matrix. Furthermore, 12 lamination arrangements were made to evaluate the effect of different fiber angles, stacking sequences, and strengths of the composite by constantly checking the jute/carbon ratio and modifying the stack design and fiber direction.

**Figure 6:**
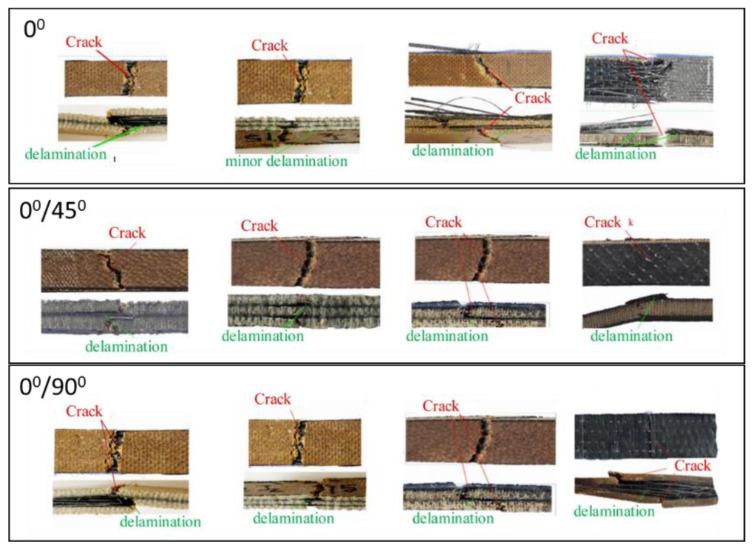
Fracture mode jute/carbon composite after tensile test with different fiber orientations (modified from [66]).

Figure 6 shows the fracture specimen after tensile test. The tensile strength decreases with the change from unidirectional orientation to (0°/45°) and (0°/90°) directions. The samples with unidirectional orientation showed the highest tensile strength compared with the other laminate variants because composites with unidirectional fibers will resist forces parallel to the fibers. Composites with unidirectional fibers can withstand high and low loads. For example, if the fibers are arranged in the 45° and 90° directions, the fibers with these orientations exhibit limited transmission of stress from the matrix.

The study conducted by Ali et al. [67] focused on the effects of different stacking sequences of carbon/jute composite mixed with epoxy resin on the mechanical properties. The specimens are CCJCC, CJCJC, and CJJJC. The three-point flexural test was conducted according to the ASTM D7264 standard with a thickness/span ratio of 1:20. The impact test was directed on three specimens that met the ASTM D7136 standard with the dimensions of 150 mm × 100 mm and a thickness of 2.2 mm. They concluded that, because of the extreme stiffness of the carbon reinforcement, the flexural strength of the samples with a massive outer carbon layer (CCJCC) was higher than those with the other sequences. The evaluation of the damage effect regions of different hybrid arrangements revealed that the impact strength decreased with the decrease in carbon mass. However, CJCJC can replace CCJCC without a considerable lack of resistance to impact force.

**Figure 7:**
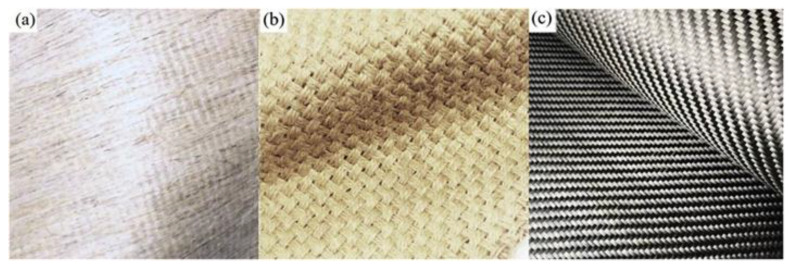
(**a**) Unidirectional jute, (**b**) woven jute fabric, and (**c**) carbon fiber fabric (modified from [68]).

The performance of NFRP and CFRP with the flexural strengthening of reinforced concrete beams was evaluated by Chen et al. [68]. Three types of fiber fabrics (i.e., unidirectional jute, woven, and carbon fibers) were used, as shown in Figure 7. The tensile test results showed that the composite consisting of two layers of carbon fiber has a tensile strength value of 2874.25 MPa, and the unidirectional jute fiber has a tensile strength value of 123.02 MPa, and the woven jute fiber has a tensile strength value of 45 MPa. Notably, two specimens, that is, six layers of woven jute with a thickness of 120 mm (7.99 kN/mm) and two layers of woven jute with a thickness of 120 mm (6.41 kN/mm), performed equivalently to the control specimen, that is, two layers of carbon fiber with a thickness of 36 mm (6.14 kN/mm) in terms of flexural stiffness. In conclusion, relying on NFRP-only materials results in a thicker structure.

### 3.3. Bamboo Fiber Hybrid Composites

#### 3.3.1. Bamboo-GFRP

**Figure 8:**
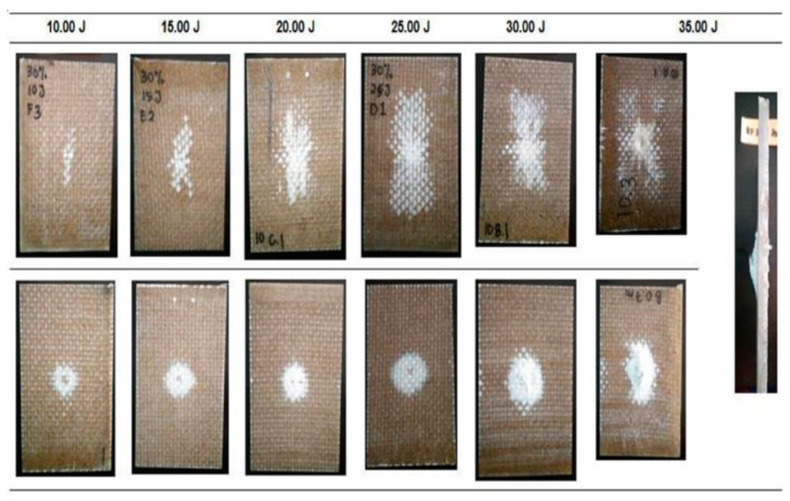
The damage on the hybrid composite after impact test with different energy levels (modified from [69]).

Bamboo fiber combined with glass fiber was studied by Shah et al. [69]. Impact testing was carried out on glass fiber/bamboo composite which was made by hand lay-up method. The test results can be seen in Figure 8. The addition of bamboo to the hybrid composite can increase the impact resistance of the composite.

Samanta et al. [70] combined bamboo/glass and jute/glass, into a hybrid composite. The layers were adjusted by modifying the direction with the angles of 0° and 90 °. According to their respective standards, each variation was tested for tensile, compressive, and flexural strengths. They focused on five samples that are; B/B/B/B, B/B/B/G, B/B/G/G, B/G/G/G, and G/G/G/G. From the tensile test showed the significant increases in tensile strength (i.e., 43.47, 72.02, 119.54, 190.94, and 340.29 MPa), tensile modulus (i.e., 6.63, 9.55, 12.65, 15.57, and 22.84 GPa), and compressive modulus (i.e., 2.85, 4.18, 5.34, 7.34, and 9.66 GPa) were observed with each addition of glass fiber layer. However, the compressive strength of the bamboo/glass hybrid is better than that of GFRP or bamboo NFRP only (i.e., 52.56, 56.19, 81.44, 64.95, and 72.00 MPa). The flexural modulus and maximum flexural stress of GFRP (i.e., 7405.93 GPa and 288.90 MPa, respectively) are relatively higher than that of its composite or natural counterpart. In summary, glass/bamboo composite reduces cost and ensures sustainability in industrial applications. Combining bamboo with GFRP and comparing their strengths are the focus of the study conducted by previous researchers [71]. Their results showed that the CBSC specimen failed differently from the bamboo composite, where the crack propagated from the bamboo fiber in the middle of the beam. Failure of the CBSC specimen was initiated by delamination of the adhesive layer between glass and bamboo fiber composite sheets because the bamboo material was still elastic during the crushing process.

#### 3.3.2. Bamboo-CFRP

**Figure 9:**
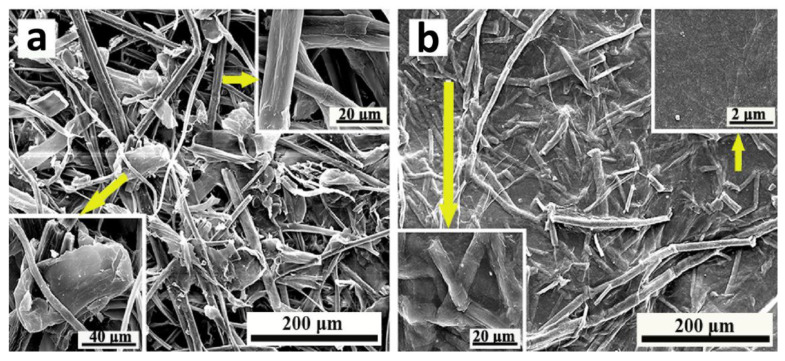
SEM images of (**a**) cellulose fibers and (**b**) freeze-dried hydrogel. The yellow arrow indicate the MBFs. (modified from [73]).

Bamboo has been widely used to make several home decorations and ornaments. The main concern here is its mechanical properties, that is, its strength and stiffness cannot meet the standards to be used as structures that can withstand heavy loads. Phong et al. [72] analyzed bamboo-fiber-reinforced composites and their strengths. Bamboo fibers were converted into micron-sized bamboo fibrils (MBFs) using micro-grinding. Bamboo fiber extraction and alkaline treatment produced composite reinforcements with unusual sizes. Bamboo is neither grass nor wood; however, bamboo has both characteristics of grass and wood. Bamboo is a renewable resource that overgrows and can be used to support building loads because of its strength. The addition of MBF to the matrix can increase the fracture toughness of the specimen. The MBF content of 0.8 wt% can increase the strength from 0.639 MPa m^1/2^ to 1.18 MPa m^1/2^. The morphology of bamboo fibers in micron size can be seen in Figure 9.

**Figure 10:**
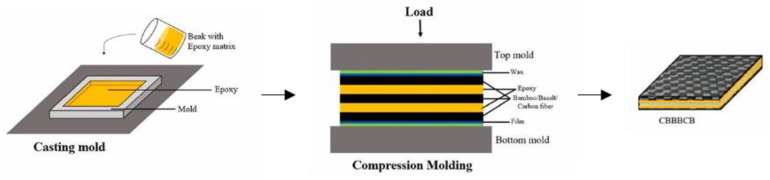
Manufacture process of carbon/bamboo fiber hybrid composite [75].

The performance of hybrid bamboo/carbon fiber composite were studied [74,75]. The composite was manufactured using the compression molding method as shown in Figure 10, the test results showed that the composite with the composition of carbon/bamboo/carbon fiber got the highest strength (90.9 MPa) than other variations. The influence of the laminae arrangement and the good interaction between the fiber and the matrix makes the composite able to withstand loads well.

### 3.4. Sisal Fiber Hybrid Composites

#### 3.4.1. Sisal/Caryota Fiber

**Figure 11:**
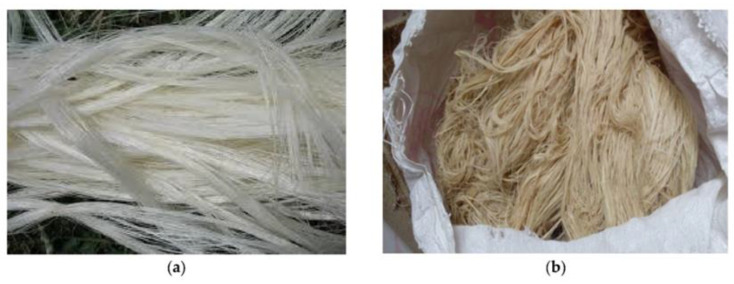
Fibers used to make hybrid composites (**a**) sisal (**b**) caryota (modified from [77]).

In general, sisal fiber has high specific strength and good absorption capability. However, sisal fiber has a limited processing temperature. This type of natural fiber is widely used in the construction industry, such as panels, doors, and shuttering plates [76]. Atmakuri et al. [77] fabricated a modified composite consist of sisal and caryota fiber as shown in Figure 11. Mechanical and moisture properties was studied. The hybrid composite of sisal/caryota get an increase in strength compared to individual fiber composites, as well as the level of water absorption that occurs by combining sisal and Caryota fibers can reduce the absorption rate.

#### 3.4.2. Sisal-GFRP

**Figure 12:**
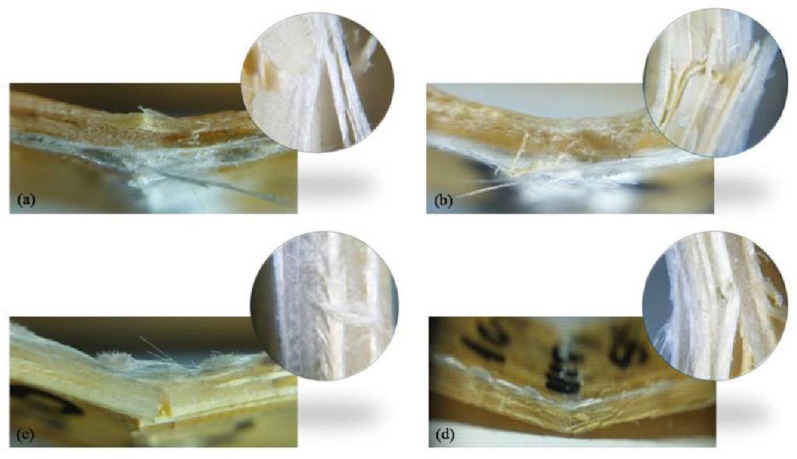
Hybrid composite fracture after flexural testing (**a**,**b**) Sisal/glass fiber composite (**c**,**d**) Glass/sisal fiber composite (modified from [79]).

Kumre et al. [78] investigated the mechanical properties of composites consisting of sisal fiber as the reinforcement, AY-105 as the matrix, and HY-951 as the hardener. The GFRP layers were located in the upper and lower parts of the sample, and the natural fiber was embedded in the intermediate layers. Sisal was combined with different volume percentages of glass fiber (i.e., 0, 5, 10, 15 and 20%). Their results showed that the sample with 20% glass fiber has the best mechanical properties compared with the other variants. Research on the bio-composite of sisal fiber combined with glass fiber was also carried out [79]. The bending test showed that the arrangement of the lamina affected the strength and damage to the composite hybrid. Figure 12 shows the sisal/glass composite hybrid after testing.

**Figure 13:**
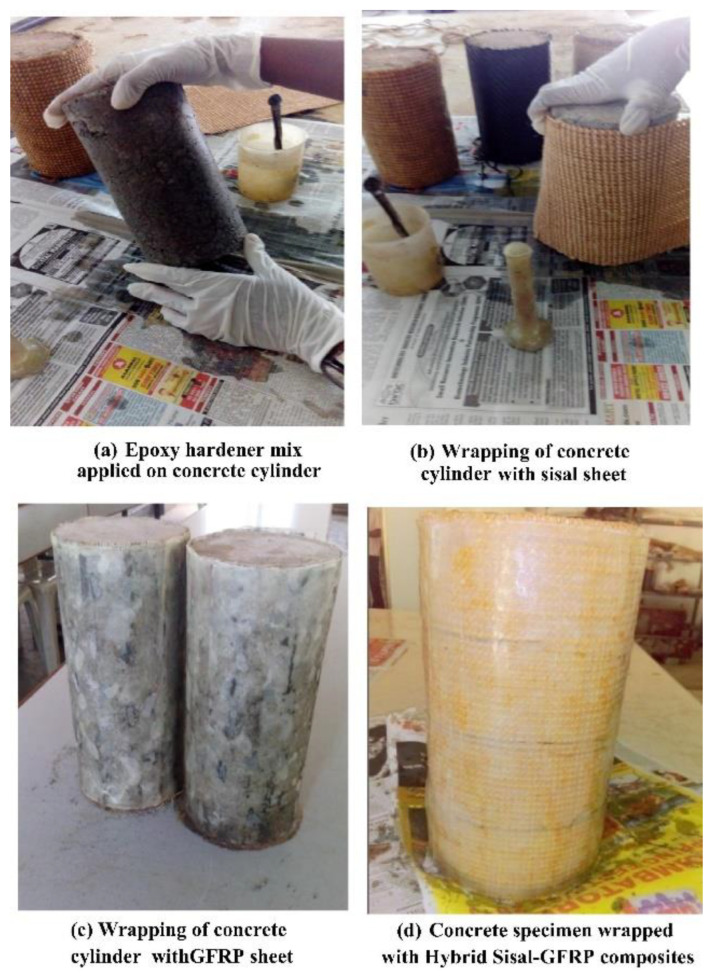
Sample manufacturing process: (**a**) coating the concrete surface with resin, (**b**) application of sisal fiber to the concrete surface, (**c**) application of glass fiber to the concrete surface, and (**d**) finished concrete that has been laminated with sisal and glass fibers (modified from [81]).

Sen and Reddy [80] reported that restoration and renovation of old and existing reinforced material structures are necessary to appropriately extend their useful life and significantly decrease maintenance costs. The strength of structures decreases because of structural degradation caused by the degradation. In the paper of Padanattil and Jayanarayanan [81], natural fibers have the potential to reinforce the matrix. However, natural fibers have low strength and durability. The study used an epoxy matrix and a hardener with a ratio of 100:15. The fibers were arranged according to the required variation with a cylindrical mold using the hand lay-up technique. Figure 13 shows the steps to manufacture the sample. Their results showed the remarkable performance of hybrid sisal/glass fiber composite compared with the CFRP and GFRP composites. The energy absorbed by GFRP is 243.85 Nm, that by CFRP is 1079.06 Nm, and that by hybrid sisal/GFRP is 1032.25 Nm. In conclusion, the combination of sisal and glass fibers as reinforcement is a good replacement option for CFRP in the ever-growing construction materials market.

Natural fibers are used to develop green materials that are fully biodegradable to solve environmental problems. Hybridization enables the adaptation of the effects of the connection according to specific conditions to achieve optimal performance. The mixture of natural and glass fibers is widely used in automotive parts. Prabhu et al. [82] combined sisal and glass fibers with tea waste and compared them to metals or alloys. Fiber-reinforced composites have significant advantages, such as high strength/weight ratio, corrosion resistance, light permeability, and ease of repair. Composites with a matrix containing 60 and 40% fiber have been fabricated. Variants are made by combining sisal and tea waste fibers.

**Figure 14:**
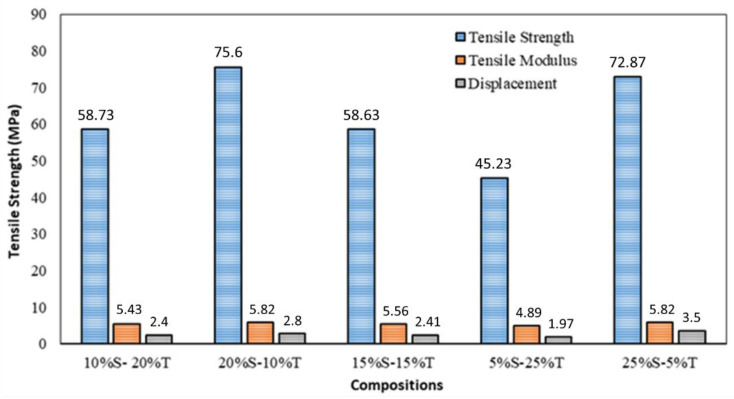
Tensile strength of composites with different consentration of fiber dan tea waste (modified from [82]).

The tensile test results shown in Figure 14 indicate that sample number 2 with 60% matrix, 20% sisal fiber, 10% tea waste fiber, and 10% glass fiber exhibited the best performance with a tensile strength of 75.6 MPa.

### 3.5. Kenaf Fiber Hybrid Composites

#### 3.5.1. Kenaf-GFRP

**Figure 15:**
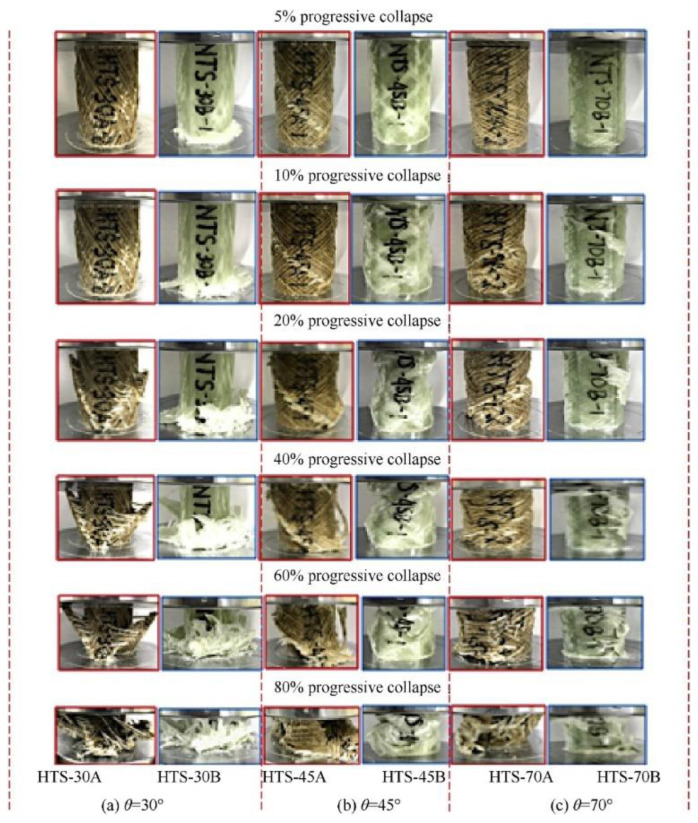
Comparison of different winding orientations of kenaf/glass fiber composite pipe and GFRP pipe (modified from [83]).

The effect of different winding directions (i.e., 30, 45 and 70°) on the impact strength of kenaf/glass fiber composite compared with that of GFRP has been studied by Supian et al. [83]. This study created a hybrid pipe made of natural/synthetic fiber composite and discovered possible applications of the hybrid pipe with diverse mechanical properties and strength performance via filament winding technology. The quasi-static axial compression test is performed according to the ASTM D7336M12 standard. Figure 15 shows each sample’s continuous compression loading process (i.e., 5–80%) with different orientations until failure occurs.

Hybrid composite pipe (HTS70A) exhibited a higher energy absorption capacity than glass fiber composite pipe. Glass fiber composite pipe has a significant capacity to improve energy absorption during quasi-static pressure loading. In contrast, kenaf/glass fiber composite pipe has a lower density than GFRP pipe. Currently, kenaf fiber is widely developed and used because it is easy to maintain, rapidly grows, and is readily available. Mechanically, this fiber has a high yield, is relatively lightweight, and exhibits excellent tension capability with a brittle fracture type. However, to ensure the rapid growth of this fiber, a large amount of water is required, considering its core’s relatively high water vapor absorption [84,85].

The strengthening and renovation of existing installations are some of the main components of the construction industry. In the study conducted by Alam and Riyami [86], 6% NaOH solution was utilized to treat the kenaf, jute, and jute rope fibers. The mechanical properties of the panels were compared with those of the jute rope sample. The kenaf and jute fiber composites exhibited higher failure loads in the flexural tests than the jute rope fiber. Overall, these findings prove the potential of natural fibers as reinforcements, thereby significantly increasing the performance of a hybrid composite.

#### 3.5.2. Kenaf-CFRP

**Figure 16:**
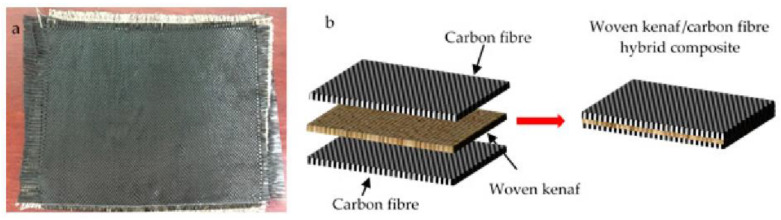
(**a**) Carbon fiber (**b**) laminae arrangement of hybrid composite (modified from [87]).

The properties of kenaf fiber make them attractive to be used as natural composites. However, the limitations of natural fiber composites are in their performance. Carbon fiber is one of the answer to the problem of strength in natural composites. Aisyah et al. [87] modified natural composite by adding carbon fiber, the specimens were made using the vacuum assisted resin infusion method with the laminae arrangement as shown in the Figure 16. The variation is in the type of woven fiber used. The results show that the plain fabric type performs better than the satin fabric. Based on observations, many voids in the composite make the strength low.

### 3.6. Flax Fiber Hybrid Composites

#### 3.6.1. Flax-GFRP

FRP materials are increasingly being applied to strengthen concrete structures. However, only a few investigations have noted the dynamic properties of GRP-reinforced concrete. Wang et al. [88] analyzed the dynamic compression properties of normal-weight concrete, flax-FRP concrete (FFRPPC), and glass-FRP concrete (GFRPPC) using a high-speed servohydraulic testing machine. The compressive strength of GFRPPC is slightly higher than that of FFRPPC. The containment efficiency of GRP is comparable to that of GRP-encased concrete under shock loads. Compared with GFRP containment, the efficiency of FFRP containment is less sensitive to the rate of stretch. The impact speed also has a limited influence on the occurrence of specimen failure.

**Figure 17:**
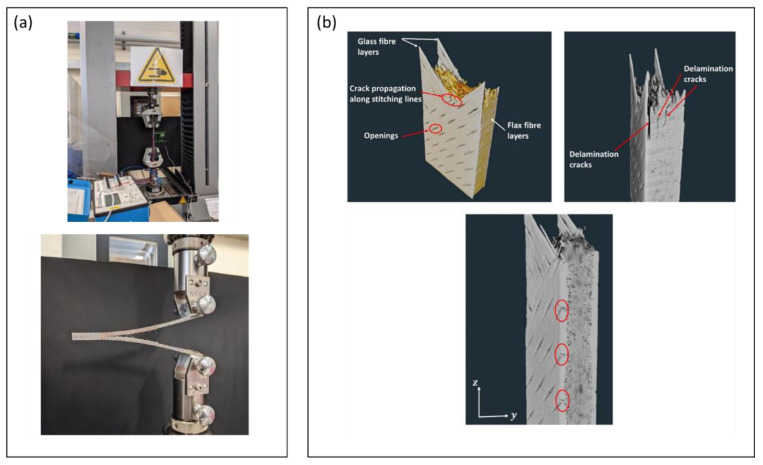
(**a**) Testing set-up (**b**) Fracture mechanism of hybrid composite [89].

Barouni et al. [89] investigated hybrid composite consist of flax and glass fiber. Tensile, DCB testing and the fracture phenomenon that occurs in the composite can be seen in Figure 17. This study focuses on the effect of hybridization of flax fiber and glass fiber on the fatigue performance of composites. Hybrid composites consisting of flax fiber and glass fiber showed more fatigue resistance than composites with flax fiber.

#### 3.6.2. Flax-CFRP

**Figure 18:**
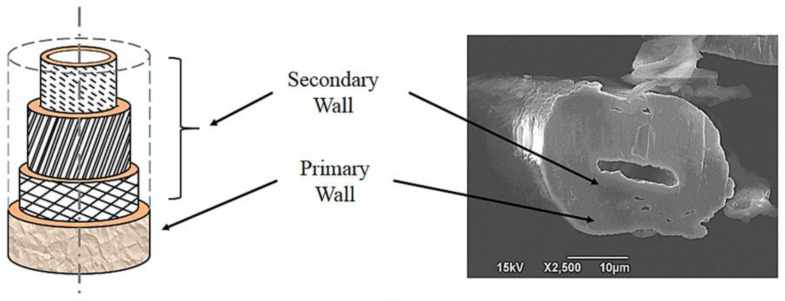
SEM of the cross section of flax fiber (modified from [91]).

Flax-FRP (FFRP) composites have attracted considerable interest because of their ecological nature and moderately good specific mechanical properties. Semicrystalline cellulose microfibrils are entrenched in flax fibers’ pectin and hemicellulose matrices. Rueppel et al. [90] reported that FFRP compounds with a given surface angle orientation were consistently dampened approximately 2 to 3 times more than CFRP compounds at low frequencies and low elongations. The attenuation of both connections increased with the increase in layer angle orientation at less than 300 Hz. The fiber also exhibited a high damping capacity of 64.4% for unidirectional FFRP composite at 1.259 Hz in the fifth vibration mode with no noticeable changes in the elastic modulus. Their study showed that FFRP compounds are both stiff and efficient at damping vibrations. However, the use of a hybrid composite can be a low-cost alternative method for addressing other issues, such as bending or tension. Figure 18 shows the cross-section of flax fiber.

**Figure 19:**
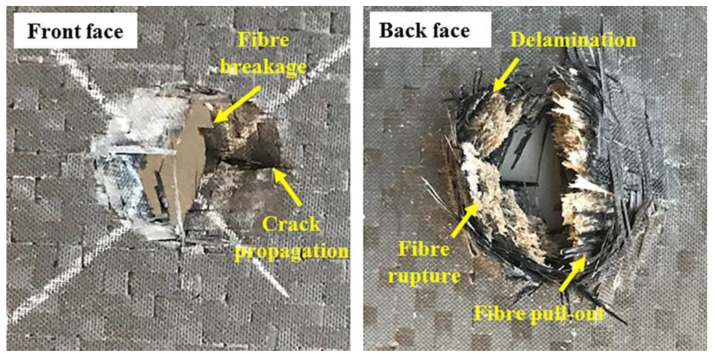
Photo images of flax/CFRP specimen failure at front and back faces (modified from [93]).

GFRP and CFRP are widely used as concrete-filled FRP tubes. However, their high initial cost meant further research needed to be conducted to find alternatives. Yan and Chouw [92] investigated the strength of different configurations of pure concrete, GFR concrete, CFR concrete, and flax-reinforced concrete composites under pure axial load. The inclusion of coconut fibers in concrete with a 1% mass fraction of 1% concrete decreases the compressive strength, whereas the inclusion of fibers with a 1% cement fraction increases the compressive strength. Their study showed that natural GRP composites and plant fibers could be used as building materials. Figure 19 shows images of flax/CFRP composite after the impact test.

Flynn et al. [94] analyzed the mechanical properties, particularly the stiffness (Young’s modulus), of synthetic hybrid fiber made from flax and carbon fiber. Then, they compared the synthetic hybrid fiber with aluminum 6061. Their test results showed that the hybrid flax fiber CFRP material exhibited superior mechanical properties to aluminum 6061, with a tensile modulus of 2%, the tensile strength of 252%, and damping ratio of 114%. This flax fiber is relatively strong and commonly used to make clothing but has low elasticity and produces a large amount of dust during the insulation process [95,96].

### 3.7. Banana Fiber Hybrid Composites

#### 3.7.1. Banana-GFRP

An example is presented by Chand et al. [97] in their paper on reinforcing epoxy using banana/glass fiber. Tensile and hardness tests were conducted on three different volume fractions (i.e., 10, 20 and 30%). Using the vacuum bag molding process, samples with banana-fiber-reinforced and banana/glass-fiber-reinforced epoxy hybrid composite laminates were successfully developed. The flexural strength of the hybrid composite containing banana/glass fiber was the highest at 162.42 MPa, which was higher than that of the hybrid composite containing banana fiber woven laminate (i.e., 44.99 ± 6.26 MPa). The hardness test results showed that the sample EBG1 exhibited the highest value of 39.65 MPa. The composite consisting of banana/glass fiber exhibited high tensile and flexural strengths but low toughness resistance.

**Figure 20:**
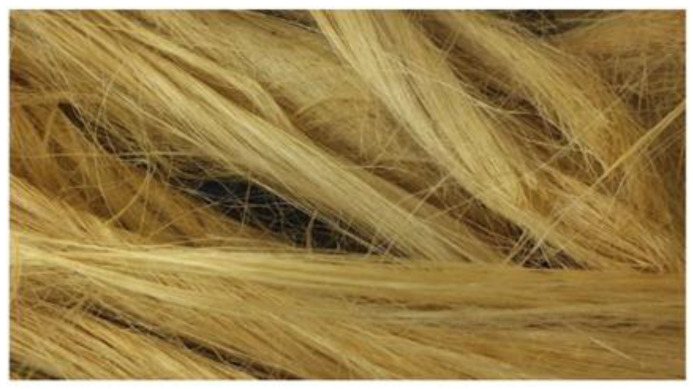
Raw banana fiber (modified from [99]).

Bhoopathi et al. [98] discussed the mechanical properties of different sequences of banana, hemp, and glass fibers. They performed the tensile test according to the ASTM D638 standard, flexural test according to the ASTM D790 standard, and impact test according to the ASTM A370 standard. Their test results indicated that hemp/banana/glass fiber exhibited the highest tensile strength of 60.99 MPa compared with glass/banana fiber, with a tensile strength of 60.45 MPa. Hemp/glass fiber showed the highest flexural strength of 1.2 KN compared with hemp/banana/glass fiber, with a flexural strength of 1.17 KN. The energy absorbed by the fibers ranges from 7.33 J to 9.33 J. In summary, the hemp/banana/glass fiber hybrid performed optimally compared with the two other options. Figure 20 shows glass and banana fibers.

#### 3.7.2. Banana-CFRP

**Figure 21:**
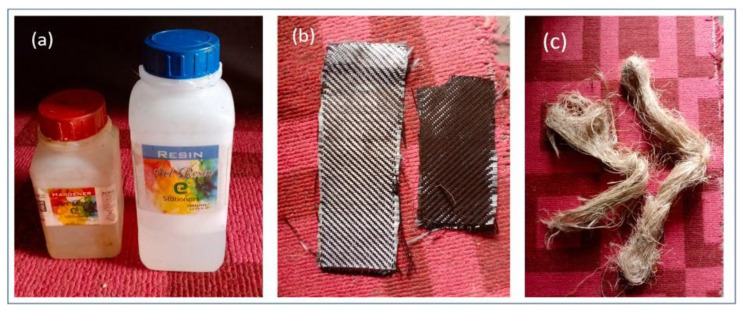
(**a**) Epoxy resin and hardener (**b**) Carbon fiber (**c**) Treated banana fiber that used in this research (modified from [100]).

Research using a combination of banana fiber and carbon fiber was conducted by Oyewo et al. [100] The composite was made using the hand lay-up method with three variations arrangement of laminae (C/C/B, C/C/B/B and C/C/B/B/B). This studied aims to determine the level of water absorption in hybrid composites. The material used can be seen in the Figure 21, where there are carbon fiber and banana fiber that has been treated previously. The ratio of epoxy resin to hardener is 2:1.

Higher natural fiber content in the composite material will automatically increase the water absorption rate. This is proven in this study, where things that affect the water absorption rate are the arrangement of the lamina, fiber treatment, and temperature. Composites with the highest number of fiber arrangements, without treatment and with a temperature of 50 °C get the highest water absorption rate.

Combinations of glass and carbon fibers are used to reinforce field hockey equipment. Rashid et al. [101] proposed new field hockey equipment materials to reduce costs and environmental impact while maintaining performance. Four different kinds of samples, with each variation having three specimens, were subjected to the three-point bending flexural test according to the ASTM D7264 standard. Their results showed improved flexural strength in the G(3)/B/G(3) composite (470 MPa) compared with G(8) or other composites.

## 4. Characteristics of Mixed Fibers

### Properties and Applications

CFRP and GFRP are used quite commonly in the industry. However, the high costs of CFRP and GFRP meant that alternatives, such as natural fiber composite materials, need to be investigated and considered their replacements. The advantages of natural fibers over glass and carbon fibers are reduced costs, reduced energy consumption, lower density, nonabrasive for devices and nonirritating to the skin, lower health risk, renewability, recyclability, and biodegradability [102].

According to Mohammed et al. [103], natural fiber is more environmentally friendly because of its easily degradable nature and does not cause environmental pollution like synthetic fibers. At temperatures as high as 240 °C, natural fiber begins to degrade. Moreover, the fiber constituents (i.e., hemicellulose, cellulose, and lignin) are degraded at various temperature levels. These fiber constituents significantly affect the thermal stability of natural fibers; thus, the concentrations of their structural constituents need to be considered. However, regardless of the advantages of natural fibers over common materials, such as their low cost, they are inferior because of their lower processing energy and temperature [104,105] and their rigidity and strength that are primarily based on the fiber loading [104]. These flaws of natural-fiber-reinforced polymers can be negated with the help of hybridization.

**Figure 22:**
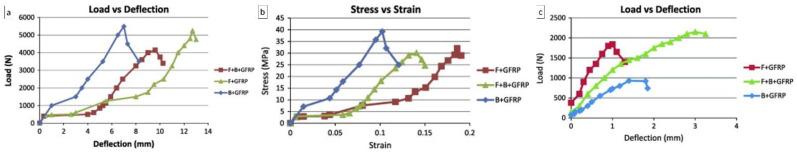
(**a**) Load vs. deflection graph of the tensile test, (**b**) stress vs. strain graph of the tensile test, (**c**) load vs. displacement graph of the flexural test (modified from [105]).

In the study conducted by Srinivasan et al. [105], a composite of glass, flax, and banana fibers is made and subjected to tensile stress test, flexural test, impact test, double shear stress, and thermal analysis. The test samples are Specimen 1 (GFBFG), Specimen 2 (GFFFG), and Specimen 3 (GBBBG). Figure 22 shows the graph of the load and deflection results of all test specimens. The experimental results show that the ultimate tensile strengths of Specimens 1, 2, and 3 are 30, 32, and 39 MPa, respectively (Figure 22b). Moreover, the ultimate flexural properties of Specimens 1, 2, and 3 are 13.54, 11.59, and 9.76 MPa, respectively.

In the previously discussed studies [95], composites were fabricated using a similar setup. Different combinations of glass, jute, and flax fibers are used to make two different test samples: Specimen 1 (GJFJG) and Specimen 2 (GJJJG). These specimens are subjected to tensile and flexural tests. The test results indicate that the ultimate tensile strength of Specimen 1 (GJFJG) is 46.5 MPa and that of Specimen 2 (GJJJG) is 56.88 MPa. The flexural strength and displacement graphs are shown in Figure 22c,d, respectively. The flexural strength of Specimen 1 (GJFJG) is 106.69 MPa and that of Specimen 2 (GJJJG) is 134.05 MPa.

Almeida et al. [106] assessed the mechanical properties of natural curaua-fiber-reinforced composite and GFRP and compared them with interlaminated hybrid composites consisting of curaua and glass fibers with different concentrations. Various tests are conducted on the samples, as shown in Table 3. The tensile test was performed according to the ASTM D3039 standard, the three-point bending test was performed according to the ASTM D790 standard, the shear test was performed according to the ASTM D5379 standard, and the short-beam steepness test was performed according to the ASTM D2344 standard.

**Figure 23:**
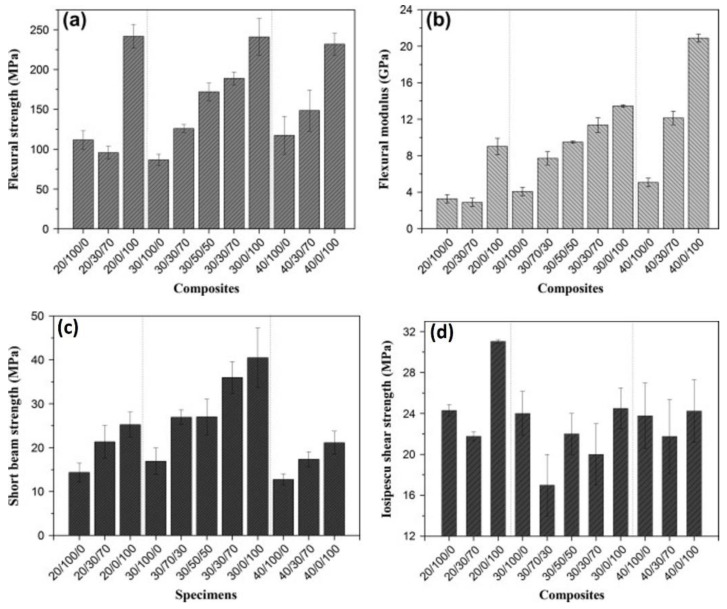
(**a**) Flexural strength (**b**) flexural modulus (**c**) short-beam strength and (**d**) Iosipescu shear strength of the composites used in the study conducted by Almeida et al. [106].

The highest bending strength was exhibited by the 20/0/100 composite consisting of glass fiber with a volume fraction of 20%, whereas the lowest bending strength was exhibited by the curaua fiber composite with a volume fraction of 30%, as shown in Figure 23a. The same trend is shown in Figure 23b, where the highest flexural modulus is exhibited by the glass fiber composite with a volume fraction of 40%, whereas the lowest flexural modulus is exhibited by the 20/30/70 composite containing 30% curaua fiber and 70% glass fiber with a volume fraction of 20%. In the short-beam strength test, the highest strength was exhibited by composites composed of glass fiber with a volume fraction of 30%, whereas the lowest strength was exhibited by curaua fiber composites with a volume fraction of 40%. The results of these tests are shown in Figure 23c. For the Iosipescu shear strength test shown in Figure 23d, the highest strength was exhibited by the 20/0/100 hybrid composite similar to that in the bending test, whereas the lowest strength was exhibited by the composite consisting of 70% curaua fiber and 30% glass fiber with a volume fraction of 30%. Moreover, Zhang et al. [107] investigated the tensile toughness and interlaminar shear of various composites made from flax and unidirectional glass fibers. The different stacking sequences shown in Table 4 are combinations of flax and glass fibers labeled as FFRP, 2G8F, 4G6F, 6G4F, 8G2F, and GFRP where the fibers were in unidirectional types.

**Figure 24:**
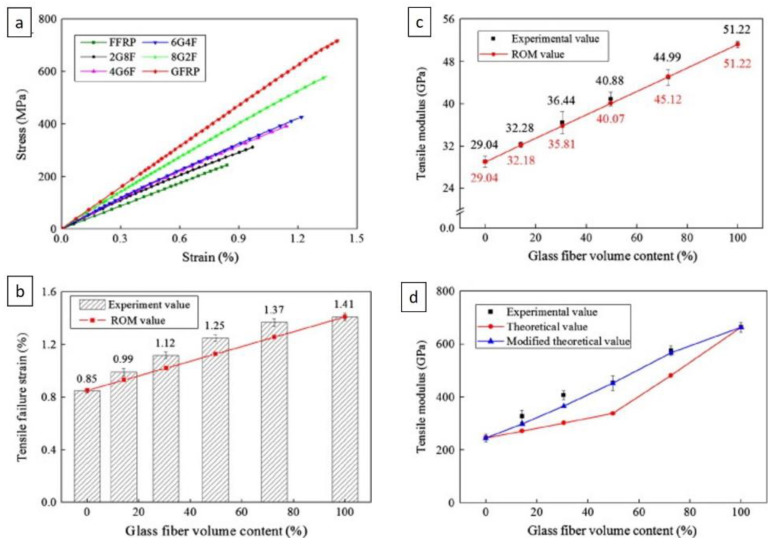
Tensile properties of flax/glass-fiber-reinforced hybrid composites with different hybrid sequences: (**a**) tensile stress–strain curves, (**b**) tensile failure strain, (**c**) tensile modulus, and (**d**) tensile modulus with different data collection methods (modified from [107]).

**Figure 25:**
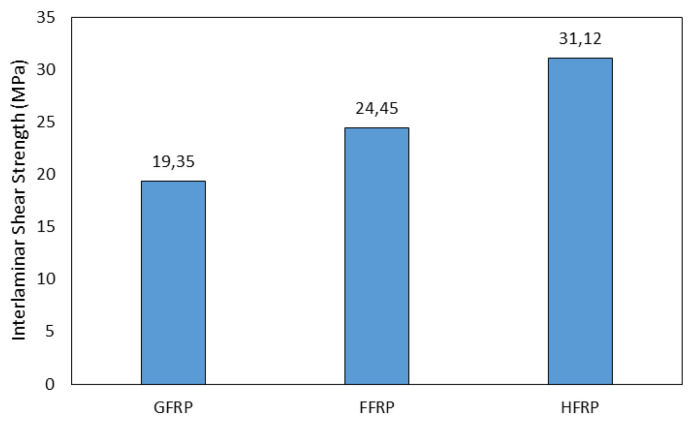
Interlaminar shear strength (glass, flax, and hybrid fiber composite) (modified from [107]).

Regarding the tensile strength behavior of the composite with different hybrid proportions, the results shown in Figure 24 indicate that the tensile properties of the unidirectional flax/glass-fiber-reinforced hybrid composites increase as the comparative volume proportion of the glass fibers increases. The stress–strain curves showed that the flax fiber composite had lower strength and lower elongation at break than GFRP. No significant difference is observed in the mechanical properties of the hybrid composites with different stacking sequences. Hybridization improves the balance of materials, enhances the interlaminar shear strength, and ensures the affordability of materials, as shown in Figure 25. The study from Hassan et al. [108] showed that they have been successfully produced a hybrid composite consisting of hessian cloth and glass fiber using the hand layup method. Table 5 and Table 6 show composites’ mechanical properties and water absorption behavior with different stacking sequences. The tensile test was performed according to the ASTM D638 standard and the flexural test was performed according to the ASTM D790 standard. The microhardness tests were conducted on the Shimadzu microhardness tester HMV-2 series.

The results shown in Table 5 reveal that hybridization improves the tensile strength, flexural strength, and hardness of the specimens. Overall, composite C4 (G/J/G/J/G) outperformed the other samples in terms of the maximum tensile strength (104.625 MPa) and flexural strength (134.65 MPa). A high jute fiber layer count in composites results in a high absorption rate, whereas a high glass fiber layer count in composites results in a high hardness value.

**Figure 26:**
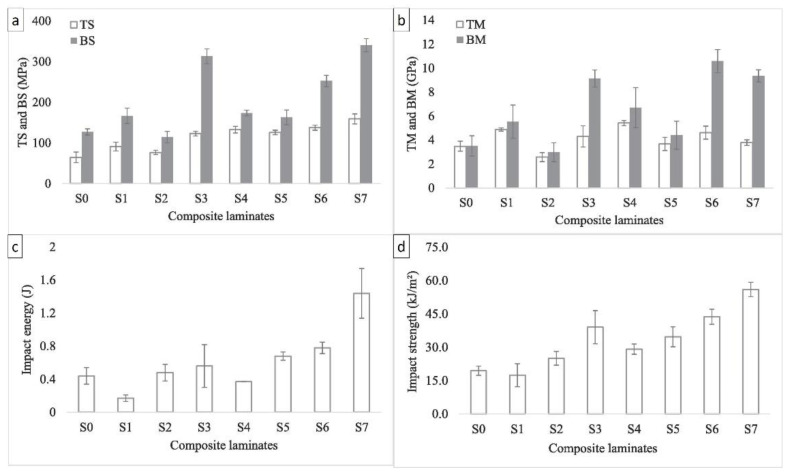
(**a**) Tensile and flexural strengths, (**b**) tensile and flexural moduli, (**c**) impact energies, and (**d**) impact strengths of the composites obtained through tests (modified from [109]).

Das et al. [109] explored the effects of fabricated composite laminates. The samples were composites consisting of jute fiber, glass fiber, and hybrid jute/glass composite with different layer arrangements. The tensile test was performed according to the ASTM D638-03 standard, the static flexural test was performed according to the ISO 14125 standard, and the impact test was performed according to the ASTM D6110-97 standard. The results shown in Figure 26 indicate that the tensile, flexural, and impact strengths of Specimen S7 were 146.3%, 167.6%, and 188.7%, respectively, higher than those of the pure jute composite (S0). Specimen S6 exhibited superior mechanical properties, 32.8% and 299.9% higher tensile and flexural moduli than pure jute composite (S0). Specimen S6 also exhibited 21.9% and 13.1% higher tensile and bending moduli than Specimen S7.

**Figure 27:**
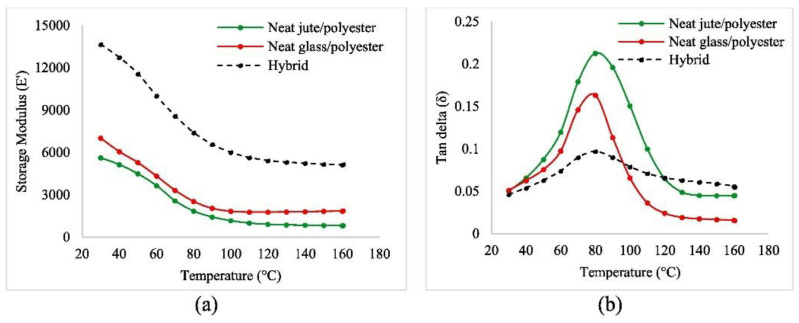
(**a**) Storage modulus (*E*′) vs. temperature and (**b**) damping (Tan*δ*) vs. temperature graphs of the composites (modified from [109]).

**Figure 28:**
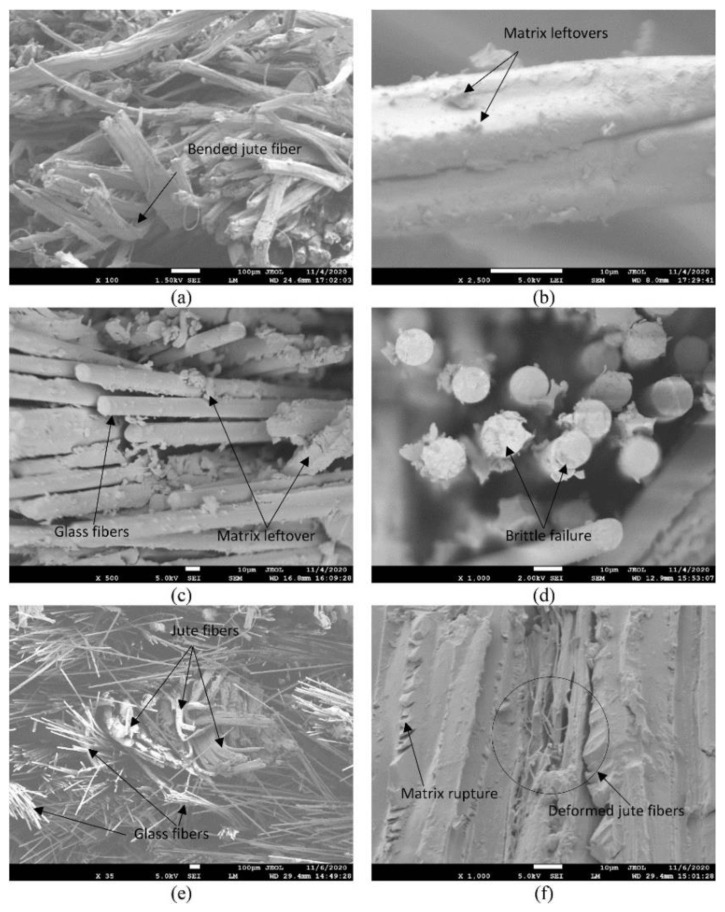
SEM images of neat jute/polyester (**a**,**b**), neat glass/polyester (**c**,**d**), and jute/glass/polyester hybrid (**e**,**f**) composite laminates at various magnifications (modified from [109]).

The hybrid composite influenced the properties of the composite. As shown in Figure 27, the hybrid composite achieved the highest damping effect compared with the other specimens, with a constant temperature. The results of the SEM observations of the surface after the tests shown in Figure 28 indicate that failure occurs because fiber pullout and cavities lead to weak interfacial bonds in the jute/polyester fiber composite. In hybrid composites, the brittle fracture phenomenon occurs because of strong fiber and matrix bonds due to the addition of glass fiber.

**Figure 29:**
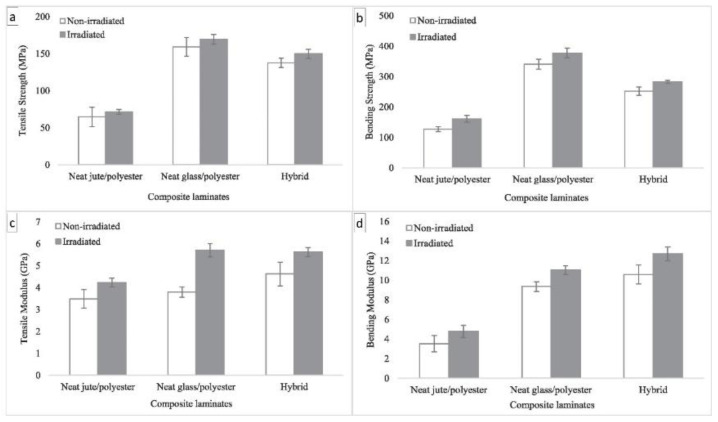
Effects of γ-radiation on the (**a**) tensile strength, (**b**) bending strength, (**c**) tensile modulus, and (**d**) bending modulus of the composites (modified from [109]).

Three different materials were exposed to an irradiation dose of 1 kGy. The results shown in Figure 29 indicate that the tensile and flexural strengths of the jute fiber composite increased by 10.7% and 26.7%, those of the glass fiber composite increased by 6.2% and 10.9%, and those of the hybrid composite increased by 8.9 and 11.9%. Similarly, in the study conducted by Sanjay et al. [110], the hybridization of materials often results in a mixture of the properties of their respective components, which could be advantageous in material design to balance manufacturing costs and mechanical performance. Some hybrid composites might exhibit unique properties, such as improved corrosion resistance, stiffness, strength, and moisture resistance. Combining two or more types of fibers could complement what the others are lacking. These properties could be further investigated and exploited in the future.

**Figure 30:**
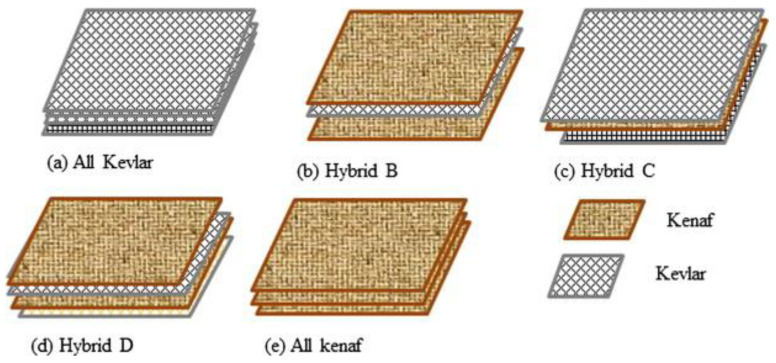
Stacking sequence of the sample composite (modified from [113]).

The effect of flax-carbon/epoxy fiber hybridization has been investigated [111] which results in an increase in impact strength, flexural and modulus. With the addition of composite flax fiber, it is proven that the damage that occurs to the composite can be minimized. This makes natural fiber hybrid composites suitable for light structural applications. Several studies have used natural fiber composites integrated with carbon or kevlar for ballistic testing purposes. Yahaya et al. [112,113] comparing the performance of the hybrid kenaf/kevlar fiber composites and kenaf fiber composites or kevlar composites as shown in the Figure 30.

**Figure 31:**
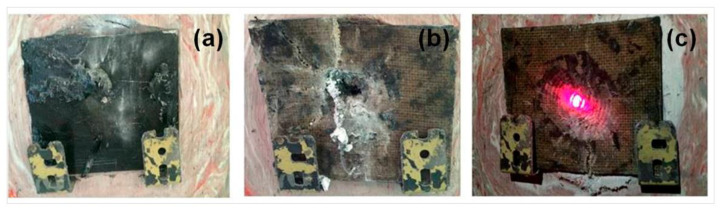
Different jute fiber percentation of the sample composite (**a**) 10% (**b**) 20% (**c**) 30% (modified from [114]).

Camposite samples manufactured using the hand lay-up technique. The quasi-static and ballistic test results showed that fiber hybridization makes the absorption of energy greater. It positively impacted the composite’s properties, which was established by the composite with Kevlar fiber on the outer layer and Kenaf fiber being kept on the inner layer (Hybrid C). The study directly described that the hybridization of kenaf and kevlar fibers could be further studied to be used as bulletproof armor. Besides kenaf, jute fiber has also been investigated to be used as a bulletproof material. The shot test that has been carried out shows that adding a higher percentage of jute fiber affects the depth of the resulting hole (Figure 31). Using jute fiber can increase the absorption of energy generated by the bullet’s impact. The resulting performance is also almost similar to kevlar [114].

## 5. Failure Behavior of Mixed Fibers

The study conducted by Shen et al. [115] showed that the addition of CFRP support to the bottom side of a parallel strand bamboo beam increases the load-carrying capacity by approximately 27%. At the same time, the reinforced beams have only slight deformations compared with the unreinforced beams. The stress on the compressive zone exhibits nonlinearity; thus, only approximately 60% of the ultimate strength of CFRP can be utilized.

**Figure 32:**
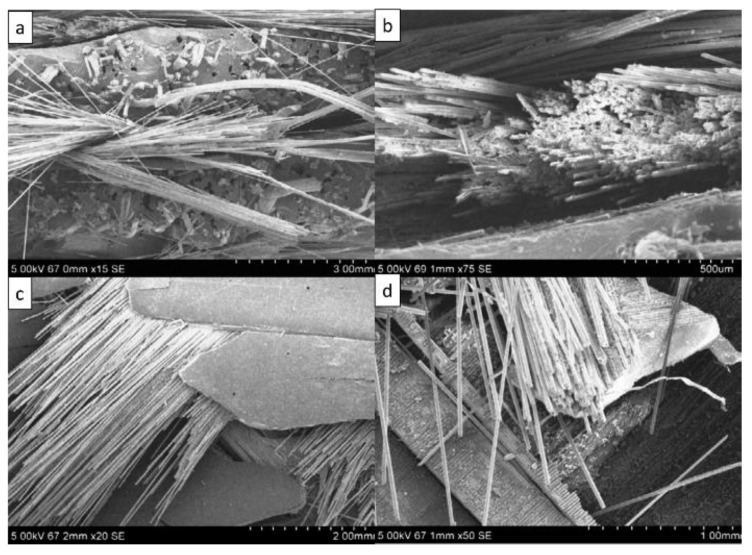
SEM images of the (**a**) sisal/glass fiber and (**b**) jute/glass fiber composites after the tensile test and (**c**) sisal/glass fiber and (**d**) jute/glass fiber composites after the flexural test (modified from [60]).

Lv et al. [116]. assessed the performance of cross-laminated bamboo (CLB) slabs reinforced with CFRP using two methods. In Method A, the CFRP slabs are pressed onto the bottom layer, whereas in Method B, CFRP is glued between layers of CLB slabs. They used the four-point bending test to determine the maximum load. Their results showed that reinforcing slabs using Method A significantly increases the load capacity of the specimen, whereas Method B negatively affects the load capacity of the slab. Senthilnathan et al. [117] reported that human hair/coconut coir/glass fiber/human hair composites have considerably better impact strength, flexibility, and double shear strength than GFRP. Previous research [60] analyzed the failure behavior of sisal/glass and jute/glass composite samples under tensile and flexural loads using SEM (Figure 32). Their results showed that composite consisting of sisal fiber and GFRP performs better in the tensile test, whereas composite jute/glass performs better in the flexural test.

**Figure 33:**
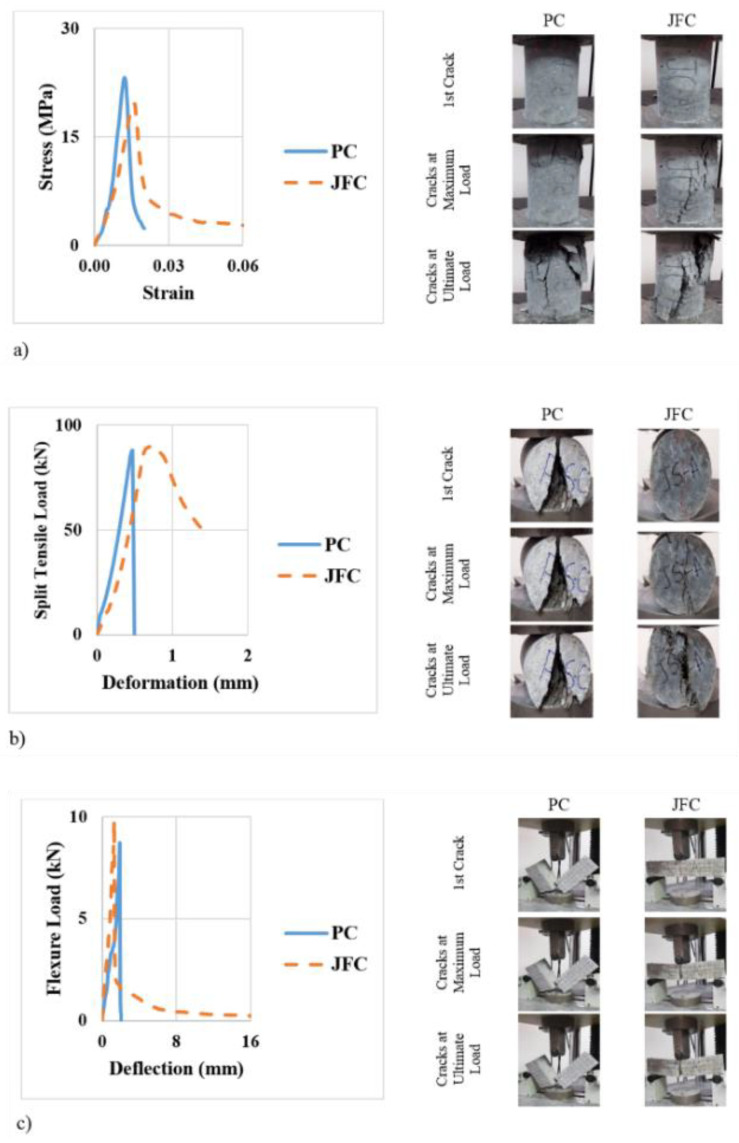
Mechanical behavior (left) and crack propagation (right) under different testing processes: (**a**) compressive, (**b**) tensile, and (**c**) flexural tests (modified from [118]).

Sabri [118] reported that plain concrete bridge piers reinforced with GFRP rebars exhibit crushing failure, whereas jute-fiber-reinforced concrete reinforced with GFRP rebars exhibits a bridging effect during failure. The failure types are shown in Figure 33 (right column).

The compressive test results shown in Figure 33 indicate that the addition of jute fiber to concrete increases its maximum energy, crack energy, total energy, and toughness index by 36.4, 260%, 143, and 85.4%, respectively, compared with that of plain concrete. This significant increase in energy dissipation and toughness is due to the crack-arresting mechanism of jute fibers. Tokoro et al. [119] showed that the addition of bamboo to annealed polylactic acid (PLA)/BF composite increases its heat resistance and thermal properties compared with annealed PLA. This condition occurred because the bamboo fibers enhance the crystallinity of PLA in the hybrid composite and the deformation constraints of PLA around bamboo fibers. The impact strength of composites that are reinforced with medium-length bamboo fibers is considerably increased because of the repeated incidence of long fiber pullouts. Khalid et al. [120] reported that epoxy/fiber composites exhibit brittle failure behavior under on-axis loading but exhibit ductile failure behavior under off-axis loading. Moreover, jute/epoxy composites exhibited mechanical properties that are inferior to those of CFRP/epoxy and GFRP/epoxy composites. Thus, they recommended using jute fiber only in hybridization with other fibers.

**Figure 34:**
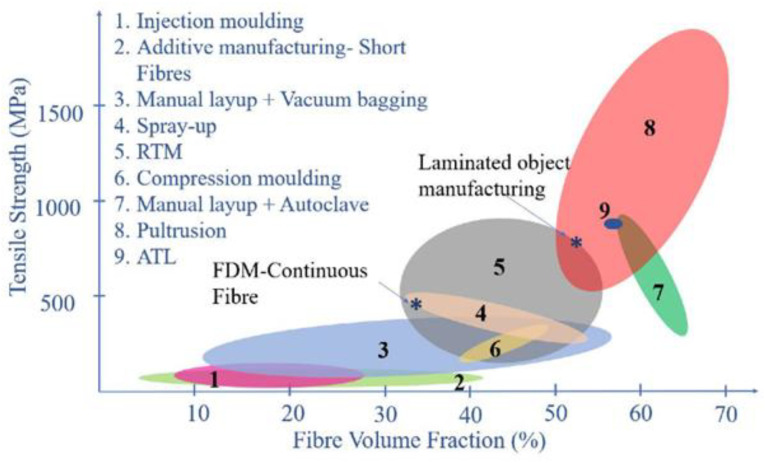
Comparison of the tensile strength and fiber volume fraction in fiber-reinforced composites (modified from [125]).

The research conducted by Capozucca [121] assessed concrete reinforced with CFRP rods mounted near the surface that was subjected to bending loads and determined that the reinforcements successfully prevented the development of cracks, as evidenced by the negligible variations in frequency values recorded during a dynamic experimental test. Meanwhile, Hassan et al. [122] analyzed the characteristics of the concrete/CFRP bonding system in a natural tropical climate with a pullout test. Their test results obtained using the Arcan method indicate that the concrete material is weaker and more prone to the effects of shear forces than epoxy adhesive materials and CFRP plates. The concrete/CFRP bonding system can transfer stress linearly and uniformly from the CFRP plate to the concrete at low loads. By contrast, at high loads, the stress occurs nonlinearly and nonuniformly, which then causes failure and local debonding of the material. Promis et al. [123] investigated the properties of concrete columns combined with FRP under compressive loads and determined that the FRP reinforcement rigidity is key to increasing loss capacity. Loss progression depends on the elasticity of the reinforcement and affects the stiffness, elasticity, and degenerate energy levels. Fully wrapped columns show rigid and solid behavior, whereas band-reinforced columns show multiple cracking around some rigid solids matching the reinforcement bands. The seismic response of the reinforced columns depends on the arrangement of the reinforcement (Young’s modulus, thickness, and spacing of bands). Zou et al. assessed the strength of FRP–concrete hybrid sections for bridge application [124]. The FRP hybrid manufacturing process dramatically affects the resulting mechanical strength, as shown in Figure 34. In this regard, the four main manufacturing processes used for custom-made FRP elements are hand layup, RTM, pultrusion, and filament winding.

Strength and damage reduction in concrete beams reinforced with jute, CFRP, and GFRP subjected to flexural load were examined [80]. Their results showed that jute, carbon, and GFRP enhanced flexural strength by 62.5%, 150%, and 125%, with filled wrapping by 25, 50 and 37.5%, respectively. The concrete beams supported with JFRP exhibited the highest deformability index, proving that JFRP has good potential to be used as a structural reinforcement material. Moreover, Katogi et al. [126] showed that the fatigue strength of unidirectional jute-yarn-reinforced PLA at 106 cycles was 55% of the maximum strength, which is nearly similar to that of GFRP. The crack tips in PLA indicate that the fatigue properties of PLA strongly affect the fatigue resistance of the sample.

Based on previous research [90] reported that FFRP composite samples are 2 to 3 times better at damping low strain and low-frequency vibrations than CFRP composites. The restraining effects of both composites increase with the increase in angle-ply coordination at less than 300 Hz. The fiber also exhibited 64.4% damping capacity for unidirectional FFRP at 1,259 Hz on the fifth type of vibration with no notable changes in the elastic modulus. Their study showed that flax composite samples are stiff and effective at damping vibrations. The bond between coconut fiber and concrete with a mass fraction of 1% decreases the compressive strength, whereas the bond between coconut fiber and concrete with a cement fraction of 1% improves the compressive strength [92].

## 6. Brief Discussion

**Figure 35:**
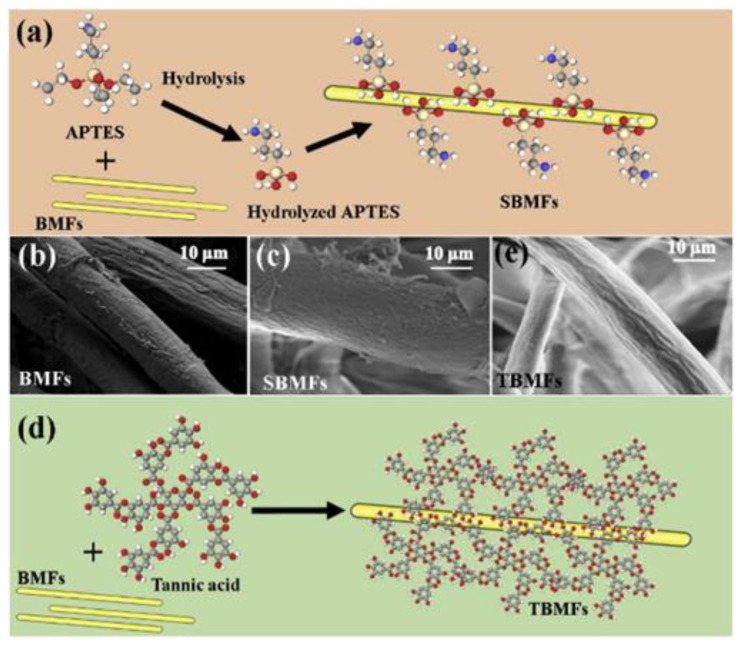
(**a**) Visualization of the combination of (3-aminopropyl)triethoxysilane and BMFs. SEM morphology of (**b**) BMFs and (**c**) SBMFs. (**d**) Visualization of the effect of tannic acid treatment on BMFs. (**e**) SEM surfaces of TBMFs (modified from [127]).

GFRP is prone to delamination because of poor matrix–fiber interaction. Researchers hope to overcome this problem by introducing nanofillers to improve the overall performance [127]. Bamboo-based cellulosic micron fiber (BMF) was released, surface treated, and embedded in epoxy resin for fiber reinforcement. On the treated BMF, the coating serves as a suitable biobased, surface-functionalized hybridization agent for GFRP. Analyses confirm the modifications in the chemical architecture of the BMF surface after silanization and tannic acid treatment. These chemical architectures are shown in Figure 35. When TBMFs were incorporated into GFRP laminates, both *σ*_y_ and *E* of the composite consisting of glass fiber increased by 48%, and the performance of the composite was improved by 55% with the addition of 1% filler by weight. Moreover, *σ*_z_ and *E*_B_ of TBMF-hybridized GFRP were dramatically increased by 71 and 103%, respectively, compared with that of GFRP. The significance of TBMFs limits the appearance of cracks in the matrix under load. In summary, natural polyphenol compounds (TA) represent a cost effective and capable surface functionalization option for natural reinforcement for use in hybrid GFRP.

The increasing demand for sustainable materials and the reduced costs of conventional materials led researchers to focus on natural-fiber-reinforced plastic composites. Natural fibers have beneficial mechanical properties that facilitate the replacement of synthetic fibers in composites [128]. Agarwal et al. [129] stated that natural fiber polymer matrix composites are manufactured using thermosetting or thermoplastic polymers. Synthetic thermoplastics with NFPRCs and natural fibers form partially biodegradable composites, equal to the percentage of natural fibers used in the composite [130].

**Figure 36:**
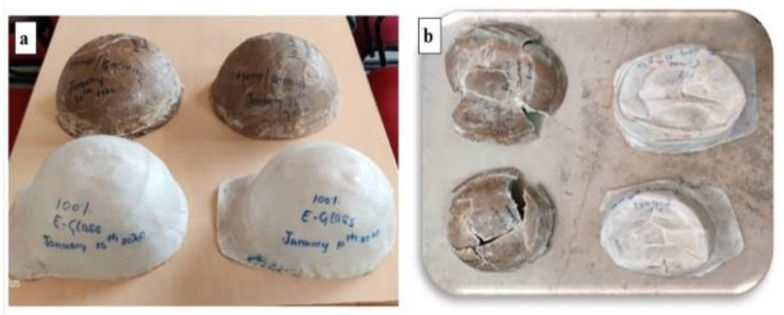
Composite helmets (**a**) before and (**b**) after the impact tests (modified from [133]).

**Figure 37:**
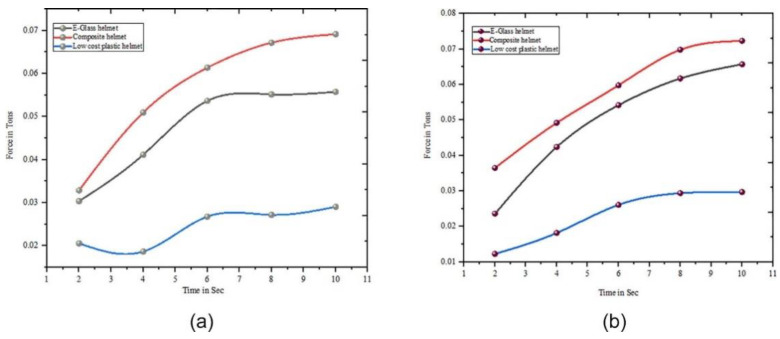
(**a**) Force vs. time and (**b**) impact energy variation vs. time graphs of composite helmets (modified from [133]).

An interesting new field of advancement is computer-aided design and material selection. Singh [131] used the hybrid intercriteria correlation and multiplicative exponent weighting optimization methods to choose materials for automotive brakes. Combinations of hemp, ramie, and pineapple fibers are made. In a similar paper, Singh et al. [132] proposed the use of the hybrid elimination and choice translating reality (ELECTRE)–entropy optimization technique to combine banana, hemp, and pineapple fibers for use in automotive brakes. Several tribological parameters, including friction recovery (%), friction performance, friction fade (%), friction stability, friction variability, wear, and friction fluctuations, influence the optimizations. The hybrid CRITIC-MEW method and ELECTRE-entropy optimization technique have been proven to be excellent optimization tools. Another example of the use of natural fibers is presented in an experiment conducted by Ravi et al. [133]. Their experiment showed that hemp/banana-fiber-reinforced plastic helmets (Figure 36) can withstand substantially higher impact force and energy compared with e-glass helmets. The impact test was conducted according to the ASTM D7136 standard, and their test results are shown in Figure 37.

**Figure 38:**
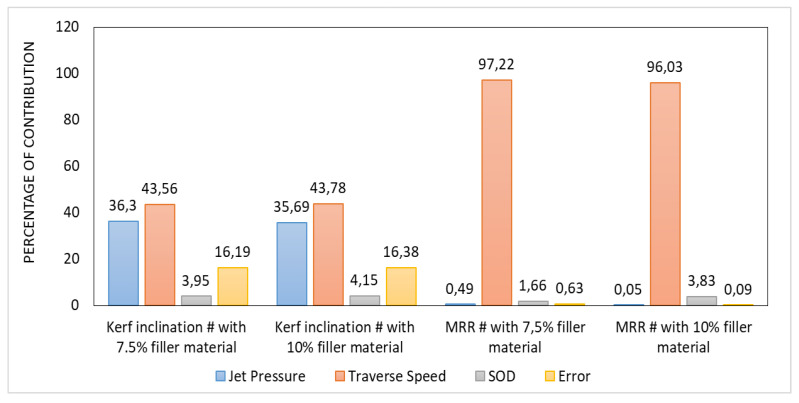
Percentage of the contribution of the water jet machining process parameters to kerf inclination and MRR (modified from [134]).

**Figure 39:**
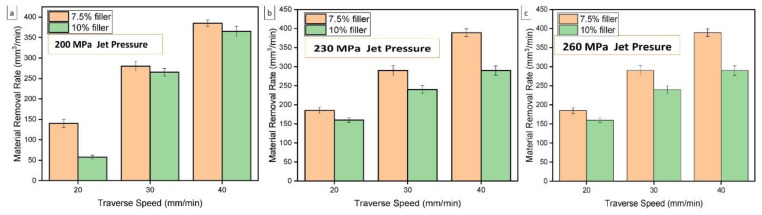
Hybrid natural fiber composite material removal rate under different pressures: (**a**) 200 MPa, (**b**) 230 MPa, and (**c**) 260 MPa, with varying traverse speeds (modified from [134]).

All composite materials made for technical applications are subjected to machining methods, such as turning, milling, and drilling. However, composite materials are difficult to control using even the most modern machining techniques. Some of the effects of machining composite materials include delamination, fiber pullouts, bulk material removal, and excessive roughness. Thus, unconventional machining methods are preferred to minimize these negative effects. One of the unconventional machining methods is Abrasive Water Jet (AWJ) machining, which has several advantages over other machining methods, including shorter fiber pullouts and machining time and negligible delamination and roughness. Jani et al. [134] analyzed the effects of varying water jet pressure, nozzle travel speed, standoff distance, and composite filler weight percentage on production yield. Their results are shown in Figure 38 and Figure 39.

Prabhu et al. [135] determined that the hydrophilic nature of natural fibers reduces the interaction between fiber and matrix, resulting in inferior mechanical properties compared with synthetic fiber composites. The interaction between fiber and matrix can be enhanced by chemical modification of the fiber [136]. Raghavendra et al. [137] treated bamboo fiber in 10% NaOH at 30 °C at a liquor ratio of 10:1 and compared it with untreated fiber. The tensile strength of the treated fiber is 46% higher than that of the untreated fiber. The buckling strength of the treated fiber is 14 MPa, whereas that of the untreated fiber is 7.27 MPa. Their results indicate significant improvements in the mechanical properties of the fibers because of the removal of hemicelluloses. Moreover, several pectins on the surface of the fibers would prevent the extraction of the fibers and improve the resistance to bending. In general, delamination is prone to occur in FRP laminates due to the lack of reinforcement along the thickness direction. Especially for the multidirectional laminate, the effect of fiber bridging on the fracture toughness is significant, which results in obvious R-curve behavior.

## 7. Conclusions

Using naturally sourced to produce composites can raise environmental awareness and reduce energy consumption compared with synthetic fibers. This paper reviewed several studies that analyzed the mechanical properties of natural and hybrid composites and their application. Based on the description that has been explained, the main discussion related to hybrid compost is described below:

1. Combining natural fiber with GFRP or CFRP is supposed to improve the mechanical properties of materials. Several studies have shown that more layers of synthetic fiber in a hybrid composite will increase its mechanical properties. Moreover, fiber treatment and characteristics directly influence the tensile, flexural, and impact strengths of hybrid composites.
2. The strength of hybrid composites is often influenced by the arrangement of the constituent fibers. The proper configuration of the arrangement to produce maximum composite strength.
3. Innovations in alternative materials highlight the possibility of utilizing natural sources, such as fiber from plants and trees, as composite reinforcement. Natural resources from trees, such as jute, flax, ramie, bamboo, and kenaf fiber, have attracted considerable attention because of their low density and biodegradability characteristics.
4. Due to their porous material, natural-fiber-reinforced composite has intrinsic advantages, such as better sound absorption and damping properties than GFRP or CFRP.
5. Some applications of hybrid natural fiber composites have been discussed. The automotive sector has examined hybrid glass/natural fiber systems and applications that influence natural fibers capabilities, such as their soundproofing properties. Natural fibers are also used in various structural and exterior applications in aircraft components, recreational equipment, and marine and building structures. Future studies must better comprehend the interactions between natural fibers and the matrix and to improve the processing techniques.

## Figures and Tables

**Table 1:** Properties of different types of natural fibers.

Fiber	Density (g/cm^3^)	Tensile Strength (MPa)	Modulus of Elasticity (GPa)	Matrix Polymer	Chemical Treatments	Reference
Abaca	1.5	430–813	31.1–33.6	Epoxy	Benzene diazonium salt	[40,41]
Bamboo	0.6–0.8	140–800	11–30	Epoxy, starch	Alkali or acid hydrolysis	[42,43]
Banana	1.4	500	12	Polyester, LDPE	Maleic anhydride treatment	[42,44,45]
Waste broom grass	0.864	297.58	18.28	Polyester	Alkali, seawater, and a combination of alkali and seawater	[46]
Coir	1.2	175	4.0–6.0	Natural rubeer	Basic chromium sulfate	[47,48]
Cotton	1.5–1.6	287–597	5.5–12.6	PP	Cationic monomeric treatment	[49]
Flax	1.5	345–1,035	27.6	PP	Alkali treatment	[44,50]
Jute	1.3	393–773	26.5	PP, SBR, nitrile rubber, epoxy, polyester, phenol–formaldehyde	Phenol–formaldehyde, melamine–formaldehyde, cardanol–formaldehyde	[48,50]
Kenaf	1.45	930	53	PLLA biodegradable polymer	NaOH	[51,52,53]
Sisal	1.45	510	28	PE, natural rubber, polyester epoxy	NaOH, isocyanate, sodium alginate, N-substituted methacrylamide	[48,50,54]
Sunn hemp		389	35	Polyester, PP		[50]
Wood flour	0.3–0.7	60–90	1.35	PE, PP, PVC, PS, polyurethane	Succinic acid, EHMA, styrene, urea–formaldehyde, acetic anhydride	[42,50,55]

**Table 2:** Stacking sequences of fibers in the composite specimens (modified from [63]).

Specimen 1 (Control)	Specimen 2 B/B/B/B/B (50%) Resin (50%)	Specimen 3 J/J/J/J/J (50%) Resin (50%)	Specimen 4 G/B/B/B/G (50%) Resin (50%)	Specimen 5G/J/J/J/G (50%) Resin (50%)	Specimen 6 G/B/J/B/G (50%) Resin (50%)	Specimen 7 G/J/B/J/G (50%) Resin (50%)
Resin Polyester	Bamboo	Jute	Glass fiber	Glass fiber	Glass fiber	Glass fiber
	Bamboo	Jute	Bamboo	Jute	Bamboo	Jute
	Bamboo	Jute	Bamboo	Jute	Jute	Bamboo
	Bamboo	Jute	Bamboo	Jute	Bamboo	Jute
	Bamboo	Jute	Glass fiber	Glass fiber	Glass fiber	Glass fiber

**Table 3:** Codes and compositions of the samples used in the study conducted by Almeida et al. [106].

Sample	Overall V*_f_* (%)	Caraua Fiber Content Ratio—C*_cu_* (vol.%)	Glass Fiber Content Ratio—C*_g_*(vol.%)
20/100/0	20	100	0
20/30/70	20	30	70
20/0/100	20	0	100
30/100/0	30	100	0
30/70/30	30	70	30
30/50/50	30	50	50
30/30/70	30	30	70
30/0/100	30	0	100
40/100/0	40	100	0
40/30/70	40	30	70
40/0/100	40	0	100

**Table 4:** Stacking sequences of composites (modified from [107]).

Label	Stacking Sequences	Volume Fraction Ratio (Flax/Glass)	Ply Number Ratio (Flax/Glass)
FFRP	FFFFFFFFFF	100/0	10/0
2G8F	GFFFFFFFFG	86/14	8/2
4G6F	GFFGFFGFFG	69/31	6/4
6G4F	GFGFGGFGFG	50/50	4/6
8G2F	GGGFGGFGGG	27/73	2/8
GFRP	GGGGGGGGGG	0/100	0/10

**Table 5:** Results of the tensile, flexural, and microhardness tests of the composite (modified from [108]).

Sample Name	Fiber Orientation	Tensile Strength (MPa)	Flexural Strength (MPa)	Hardness HV
C1	JJJJJ	38.6875	54.71	13.0
C2	JJGJJ	59.375	80.4	20.8
C3	JGJGJ	64.025	85.5	27.7
C4	GJGJG	104.625	134.65	32.2
C5	GGJGG	92.1	125.95	34.5
C6	GGGGG	106.8	176.8	39.9

**Table 6:** Water absorption results of the composites (modified from [108]).

Sample Name	Fiber Orientation	Water Absorption (%)				
		24	96	120	168	192
C1	JJJJJ	7.43	7.53	7.69	7.70	7.71
C2	JJGJJ	6.26	6.55	6.71	6.75	6.75
C3	JGJGJ	5.58	5.74	5.76	5.80	5.81
C4	GJGJG	4.41	4.88	4.96	4.98	4.98
C5	GGJGG	3.39	3.72	3.76	3.77	3.78
C6	GGGGG	2.10	2.32	2.39	2.39	2.39

## Nomenclature

CF	Carbon Fiber
GF	Glass Fiber
CCM	Conventional Compression Molding
RTM	Resin Transfer Moulding
VARI	Vacuum Assisted Resin Infusion
MPa	Megapascal
GPa	Gigapascal
LDPE	Low Density Polyethylene
PP	Polyethylene
SBR	Styrene-Butadiene Rubber
PLLA	Poly-L-Lactic Acid
NaOH	Sodium Hydroxide
PE	Polyethylene
PVC	Polyvinyl Chloride
PS	Polystyrene
EHMA	Ethyl α-Hydroxymethyl Acrylate
GFRP	Glass Fiber Reinforced Polymer
ASTM	American Standard Testing and Material
KN	Kilonewton
SEM	Scanning Electron Microscope
CFRP	Carbon Fiber Reinforced Polymer
Gsm	Grams per Square Meter
NFRP	Natural Fiber Reinforced Polymer
Mm	Milimeter
J	Joule
MBFs	Micron-Sized Bamboo Fibrils
Nm	Nanometer
FRP	Fiber Reinforced Polymer
FFRPPC	Flax Fiber Reinforced Polymer Concrete
GFRPPC	Glass Fiber Reinforced Polymer Concrete
GRP	Glass Reinforced Polymer
DCB	Double Cantilever Beam
FFRP	Flax Fiber Reinforced Polymer
ISO	International Organization for Standardization
CLB	Cross-Laminated Bamboo
PLA	Polylactic Acid
FDM	Fused Deposition Modeling
JFRP	Jute Fiber Fiber Reinforced Polymer
BMF	Bamboo-based Cellulosic Micron Fiber
TBMFs	Tannic Acid Treated Bamboo-Based Cellulosic Micron Fibers
SBMFs	Silanized Bamboo-Based Cellulosic Micron Fibers
AWJ	Abrasive Water Jet
SOD	Stand-Off Distance
MRR	Material Removal Rate
